# Magnetic targeting increases mesenchymal stromal cell retention in lungs and enhances beneficial effects on pulmonary damage in experimental silicosis

**DOI:** 10.1002/sctm.20-0004

**Published:** 2020-06-15

**Authors:** Luisa H. A. Silva, Mariana C. Silva, Juliana B. Vieira, Emilia C. D. Lima, Renata C. Silva, Daniel J. Weiss, Marcelo M. Morales, Fernanda F. Cruz, Patricia R. M. Rocco

**Affiliations:** ^1^ Laboratory of Pulmonary Investigation Carlos Chagas Filho Institute of Biophysics, Federal University of Rio de Janeiro Rio de Janeiro Rio de Janeiro Brazil; ^2^ National Institute of Science and Technology for Regenerative Medicine Rio de Janeiro Rio de Janeiro Brazil; ^3^ Rio de Janeiro Innovation Network in Nanosystems for Health ‐ NanoSAÚDE/FAPERJ Rio de Janeiro Rio de Janeiro Brazil; ^4^ Institute of Chemistry, Federal University of Goias Goiânia Goiás Brazil; ^5^ National Institute of Metrology, Quality and Technology (INMETRO) Duque de Caxias Rio de Janeiro Brazil; ^6^ Department of Medicine University of Vermont, College of Medicine Burlington Vermont USA; ^7^ Laboratory of Cellular and Molecular Physiology Carlos Chagas Filho Biophysics Institute, Federal University of Rio de Janeiro Rio de Janeiro Rio de Janeiro Brazil

**Keywords:** magnetic fields, mesenchymal stem cells, nanoparticles, pulmonary fibrosis, silicosis

## Abstract

Silicosis is a pneumoconiosis caused by inhaled crystalline silica microparticles, which trigger inflammatory responses and granuloma formation in pulmonary parenchyma, thus affecting lung function. Although systemic administration of mesenchymal stromal cells (MSCs) ameliorates lung inflammation and attenuates fibrosis in experimental silicosis, it does not reverse collagen deposition and granuloma formation. In an attempt to improve the beneficial effects of MSCs, magnetic targeting (MT) has arisen as a potential means of prolonging MSC retention in the lungs. In this study, MSCs were incubated with magnetic nanoparticles and magnets were used for in vitro guidance of these magnetized MSCs and to enhance their retention in the lungs in vivo. In vitro assays indicated that MT improved MSC transmigration and expression of chemokine receptors. In vivo, animals implanted with magnets for 48 hours had significantly more magnetized MSCs in the lungs, suggesting improved MSC retention. Seven days after magnet removal, silicotic animals treated with magnetized MSCs and magnets showed significant reductions in static lung elastance, resistive pressure, and granuloma area. In conclusion, MT is a viable technique to prolong MSC retention in the lungs, enhancing their beneficial effects on experimentally induced silicosis. MT may be a promising strategy for enhancing MSC therapies for chronic lung diseases.

## Graphical abstract

Magnetic targeting technique has emerged as a new strategy to aid delivery, increase retention, and enhance the effects of mesenchymal stromal cells (MSCs) but, so far, has not been performed in lung diseases. With the aid of magnets, magnetized MSCs remained longer in the lungs, and this was associated with increased beneficial effects for the treatment of silicosis in mice.

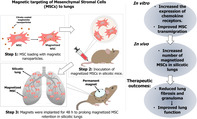

1

**Significance statement**

Although systemic administration of mesenchymal stromal cells (MSCs) ameliorates lung inflammation and attenuates fibrosis in experimental silicosis, it does not reverse collagen deposition and granuloma formation. This study established an MSC magnetization protocol using citrate functionalized magnetic nanoparticles that kept cells viable and made them magnetically responsive. With the aid of magnets, more magnetized MSCs remained in the lungs, and this was associated with enhanced beneficial effects for the treatment of silicosis in mice. Magnetic targeting may be a promising strategy for enhancing the beneficial effects of MSC‐based cell therapies for silicosis and other chronic lung diseases.

## 2 INTRODUCTION

Silicosis is an occupational lung disease caused by inhaled crystalline silica microparticles, which trigger an inflammatory response and granuloma formation in the pulmonary parenchyma, thus impairing lung function.^1^, ^2^ To date, no therapy has been able to mitigate this lung damage or reduce morbidity and mortality. Mesenchymal stromal cells (MSCs) have anti‐inflammatory, antifibrotic, antimicrobial, and antiapoptotic actions, and have demonstrated efficacy in preclinical models of a wide range of lung diseases, including silicosis.^2^, ^3^ MSCs exert reparative effects through various mechanisms: secretion of paracrine/endocrine mediators, cell‐to‐cell contact with immune cells, and transfer of organelles, such as mitochondria.^4^, ^5^

Bone marrow‐derived MSCs have demonstrated beneficial effects in preclinical studies,^6^, ^7^, ^8^, ^9^ but only modest results have been observed in clinical investigations.^10^, ^11^, ^12^, ^13^, ^14^ The reasons for this lack of efficacy in clinical trials remain unclear. Several strategies have been used to optimize MSC‐based cell therapies in an attempt to overcome this, including genetic modification and preconditioning approaches, aiming to increase MSC potency or their resistance to hostile microenvironments.^15^ An additional consideration is that, although MSCs are initially trapped in the narrow pulmonary capillaries after systemic administration, they are cleared from the lungs within approximately 24 hours.^16^, ^17^, ^18^, ^19^ This early clearance may explain why the effects of MSC administration often are short‐lasting^20^, ^21^ or may not be sufficient to promote clinical improvement in patients.^10^, ^13^

In murine models of experimental silicosis, systemic MSC administration ameliorates inflammation and fibrosis, but it does not reverse all lung histological changes, such as collagen deposition and granuloma formation.^22^ Therefore, we hypothesized that magnetic targeting (MT), a technique that is known to enable prolonged retention of MSCs in target tissues, might improve their beneficial effects in the lungs, perhaps by enabling prolonged secretion of pro‐repair growth factors directly at the site of inflammation and fibrosis. Briefly, MT involves loading the MSCs with biocompatible magnetic nanoparticles and then using a magnetic device to promote their delivery to a selected region of the body after systemic administration.^23^ MT has been shown to improve MSC retention in preclinical models of joint, spinal cord, and cardiac injuries, leading to better therapeutic outcomes.^23^ In addition, MT does not impair MSC differentiation, secretion of reparative factors, or immunomodulatory behaviors.^24^

Iron oxide (γ‐Fe_2_O_3_) nanoparticles functionalized with dimercaptosuccinic acid (DMSA) have been tested as potential agents for MT of MSCs to injured lungs.^25^ MSCs were incubated with the nanoparticles for 24 hours and then inoculated into silicotic mice. Animals were divided into two groups; in one, circular neodymium magnets (20 mm diameter, 2 mm height) were attached to the chest for up to 24 hours. At 48 hours after inoculation of MSCs, the amount of iron (from magnetic nanoparticles) in lung homogenates was significantly higher in animals in the magnet group compared to those without magnets, suggesting improved MSC retention.^25^

The present study was designed to test whether MT protocol might improve outcomes in experimental silicosis. To the best of our knowledge, the effects of MT have not yet been investigated in lung injuries.^23^, ^26^, ^27^ For this purpose, MSCs were initially labeled with superparamagnetic citrate‐capped maghemite nanoparticles.^24^ Experimental silicosis was then induced in mice and, following systemic administration of MSCs, a pair of magnets was attached to their chests for 48 hours. Comparative effects on MSC lung retention for 48 hours, as well as any potential beneficial effects of such longer retention on lung function and morphology, were assessed. In parallel, in vitro studies were conducted to investigate MSC viability and magnetic responsivity after nanoparticle loading, as well as any impacts of MT on MSC chemotactic ability.

## 3 MATERIALS AND METHODS

This study was approved by the Ethics Committee of the Carlos Chagas Filho Institute of Biophysics, Health Sciences Centre, Federal University of Rio de Janeiro, Brazil (process no. 01200.001568/2013‐87, protocol no. 024/16). All animals received humane care in compliance with the “Principles of Laboratory Animal Care” formulated by the National Society for Medical Research and the *Guide for the Care and Use of Laboratory Animals* prepared by the U.S. National Academy of Sciences.

### 3.1 Synthesis and characterization of magnetic nanoparticles

Superparamagnetic maghemite nanoparticles functionalized with citrate (γFe_2_O_3_‐Cit) were used to magnetize MSCs. The nanoparticles were prepared following protocols published by van Ewijk et al.^28^ Functionalization with citrate was performed as per the Morais et al^29^ protocol.

Total iron content in the suspensions was determined by atomic absorption spectrophotometry in a commercial Perkin‐Elmer 5000 system (Perkin‐Elmer, Norwalk, Connecticut). The content of Fe^2+^ and Fe^3+^ ions was measured colorimetrically using the 1,1‐phenantroline method. X‐ray powder diffraction data were collected by a XRD‐6000 diffractometer (Shimadzu, Kyoto, Japan). γFe_2_O_3_‐Cit crystallite size and morphology was assessed by transmission electron microscopy (TEM) (JEM 2100, JEOL, Tokyo, Japan). Hydrodynamic diameter, polydispersity index, and zeta potential measurements were obtained using a Malvern Zetasizer Nano‐ZS (Malvern Instruments, Ltd., Worcestershire, UK). Finally, magnetization of γFe_2_O_3_‐Cit was measured in a commercial Physical Properties Measurement System (PPMS, Quantum Design North America, San Diego, California) at 37°C, in the magnetic field range of −80 to 80 kOe.

### 3.2 MSC isolation and maintenance in culture

MSCs used in the present study were obtained from male C57BL/6 mouse femurs and tibias and characterized as MSCs as described elsewhere.^9^ Bone marrow‐derived MSCs were cultured in low‐glucose Dulbecco's modified eagle medium (DMEM‐LG, Gibco, Gaithersburg, Maryland) supplemented with 1% L‐glutamine (Gibco), 1% antibiotic (10 000 IU/mL penicillin and 10 000 mg/mL streptomycin, Gibco), and 10% fetal bovine serum (Gibco).^9^, ^30^ MSCs were grown under standard cell‐culture conditions (37°C, 5% CO_2_, humidified chamber). Cells from passage 3 to 6 at less than 80% confluence were used in this study.

### 3.3 MSC magnetization with γFe_2_O_3_‐Cit

Magnetization was performed by co‐culturing MSCs with γFe_2_O_3_‐Cit for 24 hours. The nanoparticles were initially diluted in DMEM‐LG supplemented with 1% L‐glutamine, 1% antibiotic, and 10% fetal bovine serum, at a concentration of 100 μg iron/mL. Nonmagnetized MSCs were cultured in the same medium, but without γFe_2_O_3_‐Cit nanoparticles. Quantitative and qualitative evaluation of γFe_2_O_3_‐Cit uptake by MSCs was performed using Prussian blue staining and TEM, as described in previous reports.^24^, ^25^

### 3.4 MSC viability after magnetization

Nonmagnetized and magnetized MSCs were then harvested by trypsinization with 1X TryPLe Express Enzyme (Gibco), and their viability was determined by flow cytometry using a live/dead viability kit (TACS Annexin V‐FITC Apoptosis Detection Kit, R&D Systems, Minneapolis, Minnesota), in accordance with manufacturer instructions. Stained cells were then analyzed by flow cytometry (FACSCalibur, Becton Dickinson, Franklin Lakes, New Jersey); each reading acquired 20 000 events. Data were analyzed using Flowing software (Version 2.5, Turku Centre for Biotechnology, Turun yliopisto, Finland). Tests were performed in triplicate.

### 3.5 MSC magnetic responsivity

Nonmagnetized and magnetized MSCs were seeded at 12‐well plates (2.5 × 10^4^ cells/well). A pair of permanent rare‐earth (NdFeB) magnets (Grade N42, diameter 12 mm, height 4 mm) was then attached to the outside bottom of the plate, exposing cells to their static magnetic fields (SMFs).^24^ The magnets were removed 24 hours after exposure, and MSCs were fixed with 4% paraformaldehyde in phosphate‐buffered saline (PBS) and Giemsa‐stained. Spatial distribution of MSCs was then analyzed using an inverted microscope (Axiovert 100, Zeiss, Oberkochen, Germany).

Magnetic responsivity of γFe_2_O_3_‐Cit‐labeled MSCs was also assessed by evaluating their transmigration in a transwell co‐culture system with silica‐activated macrophages. For this purpose, alveolar macrophages (AMs) isolated from the bronchoalveolar lavage fluid of C57BL/6 mice were used. AMs were expanded in culture with Roswell Park Memorial Institute (RPMI) medium (Gibco), supplemented with 1% antibiotic and 10% fetal bovine serum. The AMs were then seeded onto 12‐well plates (3 × 10^5^ cells/well) and cultured in the presence of silica particles (Sigma‐Aldrich, St. Louis, Missouri) (particle size 0.5‐10 μm, 100 μg silica/mL medium) for 24 hours.^31^ Nonmagnetized or magnetized MSCs were then seeded in the upper layers of hanging transwell inserts (Merck Millipore, Burlington, Vermont; 5 × 10^4^ cells/insert, 8 μm pore size) and co‐cultured with the silica‐activated AMs. In addition to chemotactic stimuli from AMs, in the lower chamber, some wells were also exposed to SMFs provided by pairs of magnets. As a negative control, MSCs were cultured without any stimuli.

MSC migration was evaluated after 15 hours. Lower surface of membrane was then fixed in 4% paraformaldehyde in PBS for 15 minutes and stained in 0.5% crystal violet for 10 minutes. The number of migrating cells was determined by counting 40 random fields per well under the microscope at ×400 magnification. Three independent experiments in duplicate were performed.

### 3.6 MT of MSCs in a murine model of silicosis

Female C57BL/6 mice aged 8 to 10 weeks were anesthetized with sevoflurane, and a 1‐cm‐long midline incision was made to expose the trachea. Silica (20 mg/50 μL saline) was then instilled intratracheally using a 31‐gauge needle. The neck incision was closed with 5‐0 silk sutures and mice returned to their cages.^32^ Fifteen days after silica instillation, animals received an intravenous injection of either magnetized or nonmagnetized MSCs (3 × 10^6^ cells/50 μL saline) or of saline (50 μL) in the internal jugular vein. MT was performed attaching a pair of circular magnets to the chest of each mouse, immediately after injection of magnetized MSCs, with the aid of a surgical tape jacket.

### 3.7 Assessment of MSC retention in the lungs

Mice were initially divided into two groups: control (n = 6) and silicosis (n = 18). After 15 days, silicotic mice were further randomized into three groups (n = 6/group): animals treated with intravenous administration of saline (50 μL); animals treated with magnetized MSCs (3 × 10^5^ cells in 50 μL saline); and animals treated with magnetized MSCs plus exposure to magnets for 48 hours. MSC retention after 48 hours was evaluated by ex vivo fluorescent imaging of the lungs and by counting iron‐loaded cells in histology, as described below.

#### 3.7.1 *Fluorescent imaging*

Magnetized MSCs were fluorescently labeled with XenoLight DiR (PerkinElmer, Inc., Waltham, Massachusetts), a lipophilic near‐infrared fluorescent dye (absorption/emission: 748/780 nm). DiR was initially dissolved in ethanol, and this solution then mixed with PBS. MSCs were incubated with 32 μg/mL DiR solution in a 37°C incubator for 30 minutes. After incubation, the cells were centrifuged and washed with PBS to remove free dye, in accordance with the manufacturer's instructions.

In vivo tracking of MSCs was then performed using an IVIS Lumina XR system (Caliper Life Sciences/PerkinElmer, Hopkinton, Massachusetts). The filters were configured at 710 nm for excitation and 760 nm for emission. Fluorescence images were acquired immediately, 24 hours, and 48 hours after cell transplantation. At 48 hours, mice were euthanized by cervical dislocation under anesthesia and ex vivo imaging of their lungs was performed. Fluorescent signal intensities (expressed as average radiance values relative to silicotic mice without MSCs) were measured and analyzed in Living Image 4.3.1 software. Two independent experiments were performed in triplicate.

#### 3.7.2 *Prussian blue staining*

Lungs were fixed with 4% paraformaldehyde in PBS and paraffin‐embedded. Sections (4 μm thick) were obtained and stained by the Prussian blue technique (1867).^33^ Briefly, the slides were washed in distilled water for 1 minute, then stained in a 1:1 solution of 10% potassium ferrocyanide and 4% hydrochloric acid for 30 minutes under gentle stirring, followed by two rinses in distilled water. Slides were counterstained in 1% Neutral Red solution for 10 minutes. Iron‐loaded cells stain blue, and were counted at ×200 magnification (four slides from each animal). The number of magnetized MSCs was then divided by the respective lung section area, measured in ImageJ software (U.S. National Institutes of Health, Bethesda, Maryland).^34^

### 3.8 Therapeutic effects of MT of MSCs in silicosis

Female C57BL/6 mice were initially assigned into two groups: control (Ctrl; 50 μL saline, i.t., n = 8) and silicosis (Sil; 20 mg silica/50 μL saline, i.t., n = 32). After 15 days, animals from the Sil group were further randomized to receive a single dose of saline (Sil‐Sal; 50 μL, i.v., n = 8), nonmagnetized MSCs (Sil‐MSC, n = 8), magnetized MSCs (Sil‐Mag, n = 8), or magnetized MSCs plus magnets for the MT technique (Sil‐MT, n = 8). Each animal received 3 × 10^5^ cells, suspended in 50 μL of saline, intravenously. Animals from the Sil‐MT group had magnets placed on their chests for 48 hours (Figure S1).

Two and seven days after saline/MSC injection, noninvasive analysis of lung mechanics was performed by whole‐body plethysmography. Nine days after saline/MSC administration, lung mechanics were analyzed invasively, mice were euthanized, and the lungs harvested for histological and molecular biology analysis (Figure S1).

#### 3.8.1 *Lung function analysis*

For *whole‐body plethysmography*, mice were placed conscious and unrestrained in single cylindrical Plexiglas chambers that were connected to a barometric whole‐body plethysmography system (Buxco Research System, Wilmington, North Carolina) for measurement of enhanced pause responses. Readings were recorded for 10 minutes and data expressed as Penh, an indirect measurement that correlates with airway resistance, impedance, and intrapleural pressure.^35^ Invasive lung mechanics analysis was performed as previously described.^21^, ^32^, ^36^

#### 3.8.2 *Histological analysis*

Left lungs of mice were fixed with 4% paraformaldehyde, and paraffin‐embedded. Slices (4 μm thick) slices were cut and stained with hematoxylin‐eosin. Then, the fractional area of the lung occupied by collapsed or normal alveoli, as well as percentage of neutrophils, mononuclear cells, and total cells in the alveolar septa, were determined by the point‐counting technique, as described elsewhere.^31^, ^37^

Collagen fiber content was quantified in lung tissue using Masson's trichrome staining.^33^ The fraction areas of collagen fiber in the alveolar septa and granuloma were determined by digital densitometric recognition in ImageJ software. Bronchi and blood vessels were carefully avoided during the measurements. Finally, lung sections stained with hematoxylin‐eosin were photographed in a microscope (Leica M205 FA, Wetzlar, Germany) to quantify the fraction area occupied by granulomas. Again, images were analyzed in ImageJ software to measure area of each granuloma and total lung area,^34^ with granuloma fraction calculated as follows:

$$\text{Granuloma fraction}\;\left(\%\right)=\frac{\sum \left(\text{Granuloma area}\right)}{\text{Lung area}}\times 100$$

#### 3.8.3 *Quantification of mediators in lung tissue homogenates*

Levels of interleukin (IL)‐1β, IL‐10 (PeproTech, Inc., Rocky Hill, Connecticut), and transforming growth factor (TGF)‐β (BD Biosciences, San Jose, California) were quantified by enzyme‐linked immunosorbent assay (ELISA) in lung tissue homogenate as instructed in the manufacturer's protocol, and normalized to the total protein content quantified by Bradford's reagent (Sigma‐Aldrich). Lung tissue was homogenized in lysis buffer (1X PBS, 0.01% Triton X, 1X Roche protease inhibitor cocktail [Roche Diagnostic, Mannheim, Germany]) using a bead mill (TissueLyser II, Quiagen, Hamburg, Germany) with a 3‐mm stainless steel bead (time: 5 minutes; frequency: 50 oscillations/s).

### 3.9 Quantitative RT‐qPCR

Reverse transcription followed by quantitative polymerase chain reaction (RT‐qPCR) was performed to measure mRNA expression in MSCs and in lung tissue homogenates. For this purpose, cells/tissues were lysed for RNA extraction through the ReliaPrep RNA Miniprep System (Promega Corporation, Madison, Wisconsin) in accordance with manufacturer recommendations. The total RNA concentration and purity was measured by spectrophotometry in a Nanodrop ND‐1000 system (Thermo Fisher Scientific, Waltham, Massachusetts). A260/A230 and A260/A280 ratios approximately equal to 2 were considered ideal for RNA purity. First‐strand cDNA was synthesized from 1 μg RNA using a high‐capacity cDNA reverse transcription kit (Thermo Fisher Scientific). Relative mRNA levels were measured by SYBR Green detection (Promega) in a PCR Mastercycler ep Realplex system (Eppendorf, Hamburg, Germany). All samples were measured in triplicate. The relative level of each gene was calculated as the ratio of the study gene to the control gene (acidic ribosomal phosphoprotein P0, *36B4*) and given as the fold change relative to samples from the control group.

The following primers were used in this study: C‐C chemokine receptor type 2 (CCR2), C‐X‐C chemokine receptor type 4 (CXCR‐4), Integrin α4, Monocyte chemoattractant protein‐1 (MCP‐1), Procollagen type I, Procollagen type III, and stromal cell‐derived factor 1 (SDF‐1). Primers 5′‐3′ sequences are presented in Table S1.

### 3.10 Statistical analysis

Statistical analyses were performed in GraphPad Prism version 6.0 (GraphPad Software, San Diego, California). First, the normality of the data was tested using the Shapiro‐Wilk test; then, the ROUT test was performed to identify outliers. If data were normally distributed, Student's *t* test, one‐way analysis of variance (ANOVA) (followed by Tukey's test), or two‐way ANOVA (followed by Bonferroni's test) were used. If the assumption of normality was rejected, the Mann‐Whitney test or Kruskal‐Wallis test (followed by Dunn's test) was used. Differences were considered statistically significant at *P* < .05.

## 4 RESULTS

### 4.1 Characterization of γ‐Fe_2_O_3_‐citrate nanoparticles

X‐ray diffraction data showed that the crystalline phase of the iron oxide nanoparticles has a cubic spinel structure (Figure S2A). This finding, taken together with the Fe^2+/^Fe^3+^ molar ratio of approximately 0.05, characterizes the magnetic nanoparticles as maghemite (γ‐Fe_2_O_3_). TEM photomicrographs (Figure S2B,C) show nanoparticles polydisperse in size in the range of 1.7 to 11.5 nm, with a predominantly cubic morphology. According to atomic absorption spectrophotometry data, the γ‐Fe_2_O_3_‐citrate nanoparticle solution used in this study contains 9 mg iron per mL, which corresponds to 12.8 mg maghemite per mL (Table 1). When dispersed in water, γ‐Fe_2_O_3_‐cit nanoparticles present an average hydrodynamic diameter of 89.6 ± 4.2 nm and a polydispersity index of 0.25 ± 0.01 nm; their size increased when dispersed in cell culture media (Table 1). Hydrodynamic diameter distributions of γ‐Fe_2_O_3_‐cit nanoparticles in both conditions are presented in Figure S2D. The γ‐Fe_2_O_3_‐cit zeta potential was −52 mV, provided by the citrate ions bound to the nanoparticle surface, and the saturation magnetic moment was 40.4 emu/g (Table 1 and Figure S2E).

**TABLE 1:** Characterization of γ‐Fe_2_O_3_‐citrate nanoparticles

Formulation	Iron content (mg/mL)	Hydrodynamic diameter (in water, nm)	Polydispersity index (in water)	Hydrodynamic diameter (in cell culture media, nm)	Zeta potential (mV)	Saturation magnetic moment (emu/g)
Citrate‐coated maghemite (γ‐Fe_3_O_2_) nanoparticles	9	89.6 ± 4.2	0.25 ± 0.01	274.3 ± 22.5	−52 ± 8.15	40.4 ± 0.01

*Note:* Hydrodynamic diameter, polydispersity index, and zeta potential data refer to average and SD of three independent samples.

### 4.2 MSCs take up γ‐Fe_2_O_3_‐citrate nanoparticles without losing viability

**FIGURE 1:**
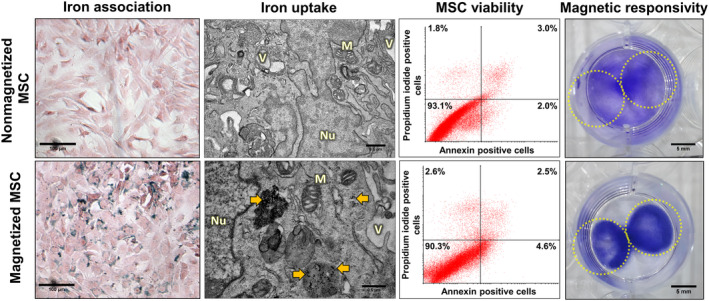
MSC magnetization with γ‐Fe_2_O_3_‐Cit. After incubation with γ‐Fe_2_O_3_‐Cit (24 hours, 100 μg/mL), intracellular iron was observed in magnetized MSC cytoplasm (stained blue on Prussian blue photomicrographs or as electron‐dense dots in transmission electron microscopy photomicrographs, yellow arrows). Cell viability after magnetization was evaluated by annexin/propidium iodide staining; the percentage of viable cells is shown in the lower left quadrants. Magnetic responsivity of magnetized MSCs was evaluated by analyzing cell distribution in cell‐culture plate wells with paired magnets on the bottom (position represented by dashed yellow circles) after 24 hours of exposure to their magnetic fields. M, mitochondria; MSC, mesenchymal stromal cell; Nu, nucleus; V, electron‐lucent vesicles

After incubation with γ‐Fe_2_O_3_‐cit nanoparticles, iron was observed in the cytoplasm of magnetized MSCs, as shown by Prussian Blue staining (Figure 1). This was not observed in nonmagnetized MSCs. TEM photomicrographs confirmed γ‐Fe_2_O_3_‐Cit uptake by MSCs, as evidenced by the presence of electron‐dense signals within vesicles (large areas) or free in the cytoplasm (small areas), corresponding to the region rich in iron oxide (Figure 1, yellow arrows). Nonmagnetized MSCs did not exhibit these morphological changes.

The viability of magnetized and nonmagnetized MSCs was evaluated using annexin/propidium iodide staining. No significant differences in the percentage of viable cells were observed between the experimental groups (Figure 1; Table S2).

### 4.3 γ‐Fe_2_O_3_‐cit conferred magnetic susceptibility to MSCs

To test whether γ‐Fe_2_O_3_‐cit‐loaded MSCs developed magnetic responsivity, they were initially seeded into culture plates containing a pair of magnets in the bottom. After 24 hours, mostly magnetized MSCs settled down in magnetic pole regions (Figure 1, yellow circles), unlike nonmagnetized MSCs, which were randomly distributed in the well. This property was also observed by approaching a magnet to a magnetized MSC suspension. The movement of MSCs in response to the presence of a magnet creating an SMF was visible (Video S1).

### 4.4 MT improved MSC transmigration in vitro

**FIGURE 2:**
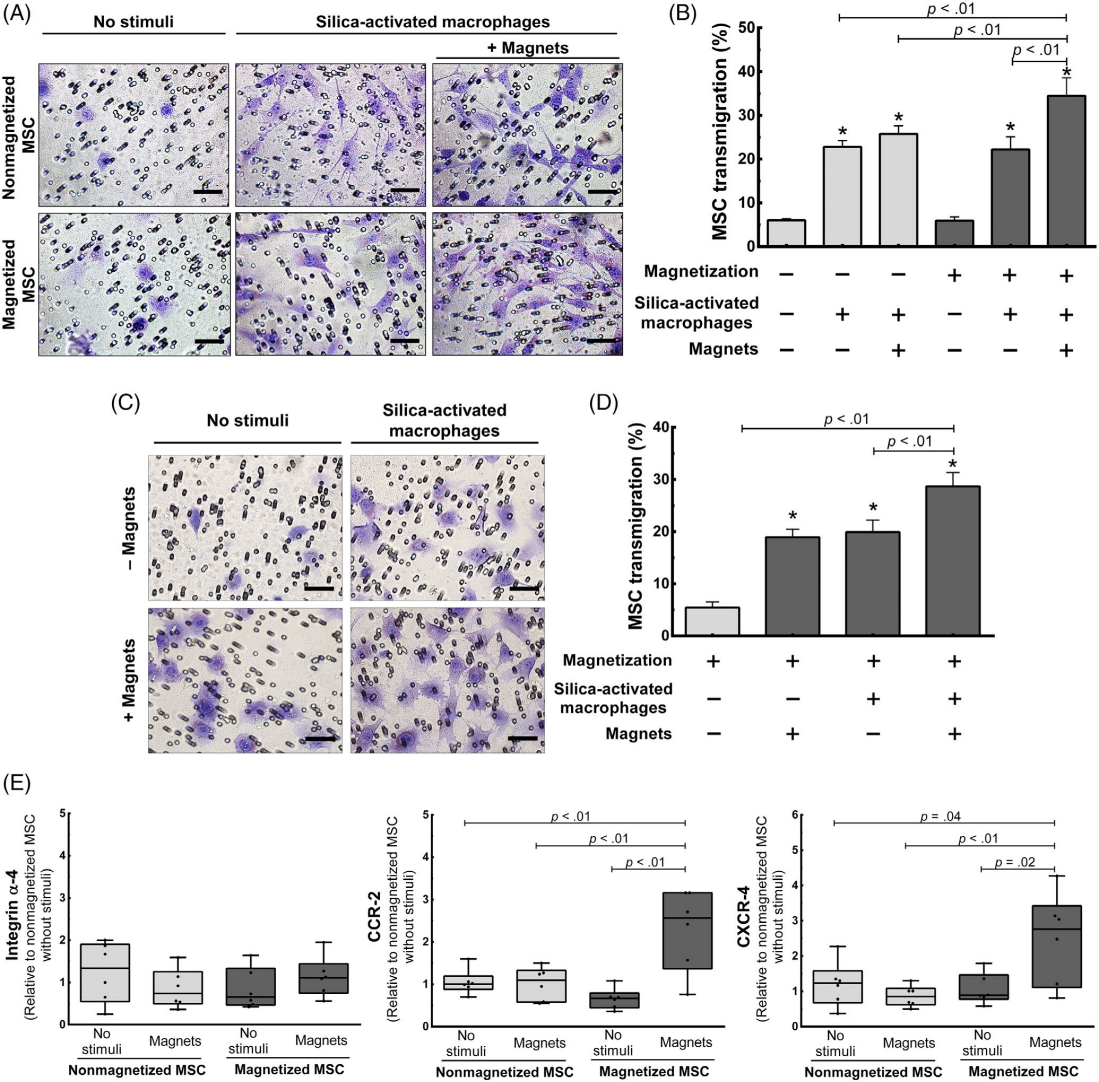
MSC transmigration in a transwell co‐culture system with silica‐activated macrophages. A,C, Representative photomicrographs of transmigrated MSCs after 15 hours of culture with different stimuli. Scale bars = 50 μm. B,D, Evaluation of MSC transmigration. Data are means + SD of percentage of transmigrated MSCs. *MSC transmigration was significantly higher (*P* < .05) in all groups co‐cultivated with silica‐activated macrophages when compared to nonmagnetized or magnetized MSCs without activated macrophages. E, Expression of α4 integrin, C‐C chemokine receptor type 2 (*CCR2*), and C‐X‐C chemokine receptor type 4 (*CXCR4*) in MSCs. Boxes show the interquartile range (25^th^‐75^th^ percentile), whiskers encompass the range (minimum‐maximum), and horizontal lines represent median values of fold change normalized to nonmagnetized MSCs without magnets (n = 6). MSC, mesenchymal stromal cell

We then investigated whether this magnetic responsivity was able to improve transmigration of MSCs when exposed to silica‐activated AMs (Figure 2). Using a transwell system, magnetized and nonmagnetized MSCs were co‐cultured with AMs, which had been previously incubated with crystalline silica microparticles. In addition to the chemotactic stimulus provided by the activated macrophages, some MSCs were also exposed to SMFs from the magnets. As a result, we found that magnetized MSCs, when additionally exposed to magnets, presented a significantly higher transmigration percentage compared to all other tested conditions (Figure 2A,B). Importantly, this condition—MSC magnetization followed by exposure to magnets—is the one that simulates MT.

Additionally, we hypothesized whether the magnetic force itself would be capable of pulling MSCs across the membrane, enhancing their transmigration. Therefore, in a second experiment, we used magnetized MSCs only, which were exposed to activated AMs, magnets, or a combination of the two (Figure 2C,D). MSCs exposed to magnets presented the same rate of transmigration when compared to those exposed to activated AMs. In parallel, magnetized MSCs exposed to both stimuli exhibited higher transmigration compared to the other experimental conditions (Figure 2D).

We then assessed whether SMFs from magnets have an impact on the expression of genes related to MSC adhesion and chemotaxis (Figure 2E). For this purpose, we analyzed mRNA expression of α4‐integrin, CCR2, and CXCR‐4 in magnetized and nonmagnetized MSCs exposed or not to magnets in vitro. RT‐qPCR data showed no alterations in α4‐integrin gene expression, regardless of the condition tested. Otherwise, magnetized MSCs exposed to magnets had significantly increased expression of *CCR2* and *CXCR4* when compared to all other conditions (Figure 2E).

### 4.5 MT increased the number of MSCs in silicotic lungs 48 hours after injection

**FIGURE 3:**
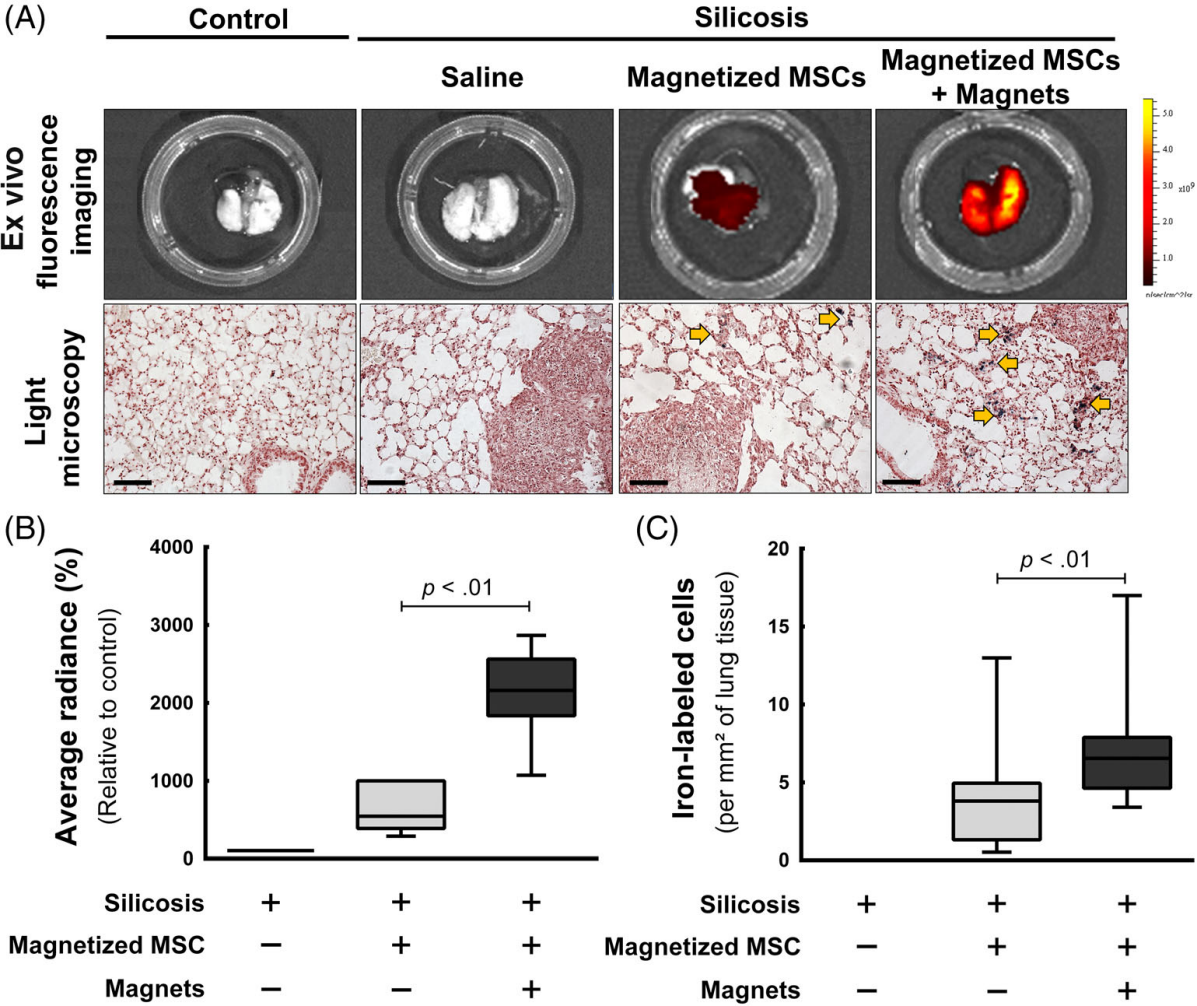
Assessments of MSC amount retained in the lungs at 48 hours. A, Representative photos of ex vivo fluorescent imaging and representative photomicrographs of lung histologic slides. Slides were stained with Prussian Blue and counterstained with Neutral Red; iron‐labeled cells are shown in blue (yellow arrows). Scale bars = 200 μm. B, Fluorescence measurement in lungs. Boxes show the interquartile range (25^th^‐75^th^ percentile) while whiskers encompass the range (minimum‐maximum) of average radiance detected in the lungs (n = 6). C, Iron‐labeled cell counting in lung histological slides. Boxes show the interquartile range while whiskers encompass the range (minimum‐maximum) of iron‐positive cells amount per mm^2^ (n = 6). MSC, mesenchymal stromal cell

Signals from magnetized MSCs stained with XenoLight DiR tracer were detected in all injected silicotic mice, at all time points, by in vivo fluorescent imaging (Figure S3). Ex vivo fluorescent imaging of lungs was performed 48 hours after magnetized MSC injection (Figure 3A). Lungs of mice injected with magnetized MSCs and subjected to placement of magnets on the chest for 48 hours exhibited significantly higher fluorescence intensities (expressed as average radiance) compared to animals that did not receive magnets (Figure 3A,B). Lungs from silicotic mice treated with saline showed no fluorescent signal (Figure 3A). Histological analyses of these lungs, stained with Prussian Blue, corroborate ex vivo fluorescence imaging data (Figure 3A,C). Mice treated with magnetized MSCs and with magnets attached exhibited a significantly higher number of iron‐labeled cells (in blue) per area compared to animals that did not receive magnets. These iron‐labeled cells were not observed in control or silicotic animals receiving saline, suggesting that the iron observed in these images came from γ‐Fe_2_O_3_‐cit (Figure 3A).

### 4.6 MT improved lung mechanics and reduced granuloma area in silicotic animals

We then evaluated whether the improvement in MSC retention promoted by MT would lead to better therapeutic effects in experimental silicosis. Silicotic mice were treated with saline, nonmagnetized MSCs, magnetized MSCs, and magnetized MSCs with external magnets, the latter henceforth referred to as MT (Figure S1). Forty‐eight hours after MSC administration, animals from all groups underwent full‐body plethysmography to evaluate lung function (Figure S4). Only the Silicosis‐nonmagnetized MSC group did not present significant differences in Penh index compared to control. On the other hand, the Silicosis‐MT group initially exhibited a higher Penh index at day 2; however, the index had decreased to control values by day 7. Last, the Silicosis‐magnetized MSC group presented significantly different Penh index values compared to control at both time points (Figure S4).

**FIGURE 4:**
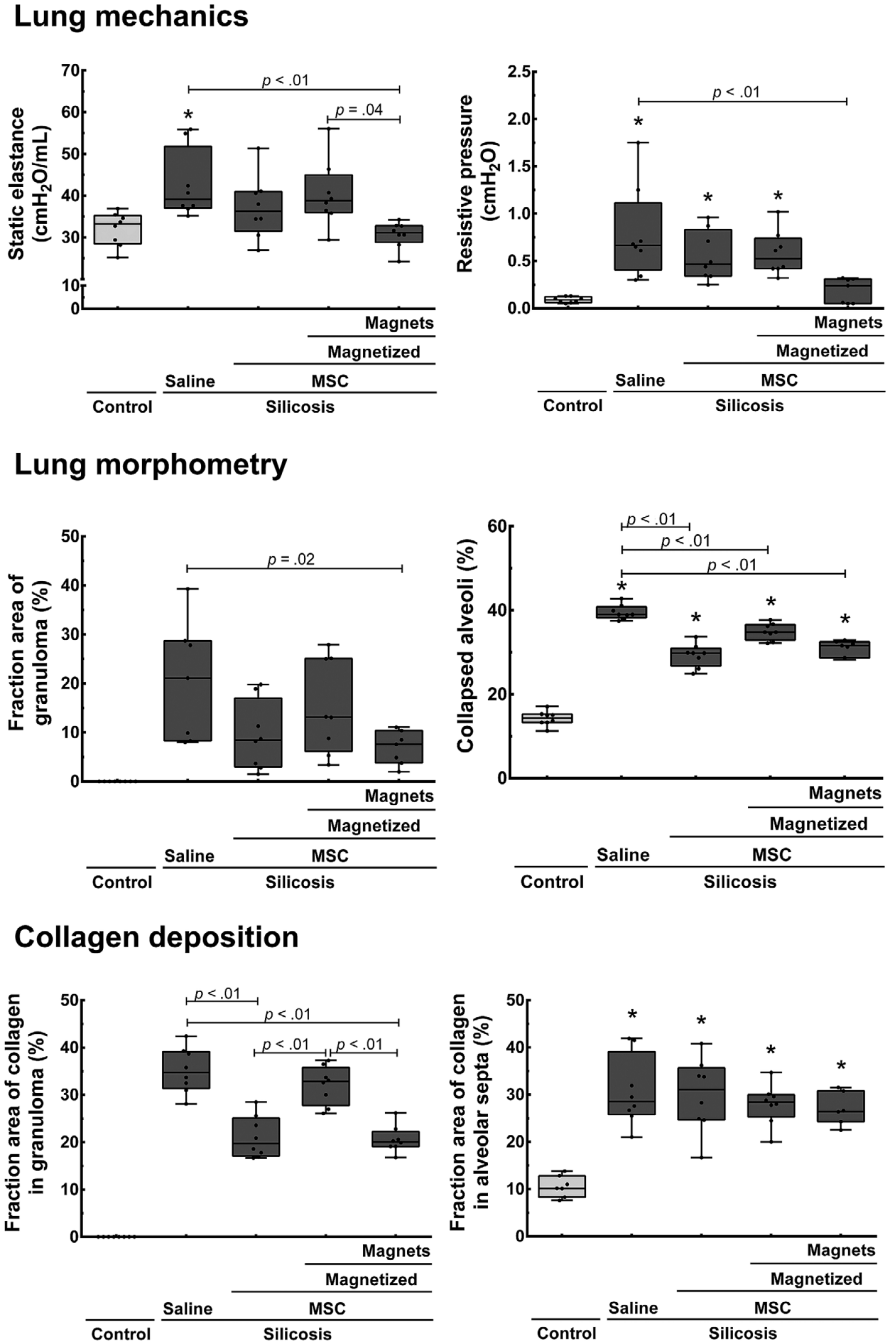
Effects of magnetic targeting on lung mechanics (upper panels), lung morphometry (middle panels), and collagen deposition (bottom panels). Boxes show the interquartile range (25^th^‐75^th^ percentile), whiskers encompass the range (minimum‐maximum) of static lung elastance, resistive pressure, granuloma area, alveolar collapse, and collagen fibers in the granuloma and alveolar septa (n = 8)

Nine days after MSC injection, lung function was measured through invasive mechanical ventilation (Figure 4). MT significantly reduced static lung elastance when compared to the Silicosis‐Saline and Silicosis‐Magnetized MSC groups (Figure 4). Although animals in the Silicosis‐Nonmagnetized MSC group also showed a reduction in static elastance, this difference was not significant (*P* = .46). Resistive pressure was reduced after treatments with nonmagnetized or magnetized MSC; however, Silicosis‐MT animals were the only group in which the difference was significant when compared to Silicosis‐Saline animals (Figure 4).

**FIGURE 5:**
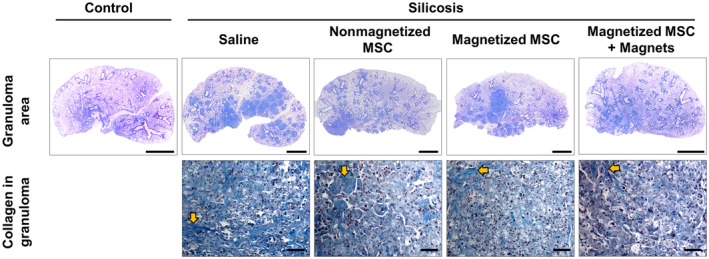
Representative photomicrographs of lung parenchyma stained with Masson's trichrome, showing collagen fibers in blue (yellow arrows). Scale bars = 2 mm and 100 μm, respectively. Note the reduction in the area of the granuloma in the Silicosis‐Magnetized MSC + Magnets (Sil‐MT) group. MSC, mesenchymal stromal cell; MT, magnetic targeting

Morphological parameters that could potentially contribute to static lung elastance reduction, such as collagen deposition, alveolar collapse, and fraction of area occupied by granuloma, were then evaluated (Figures 4 and 5). The Silicosis‐Nonmagnetized MSC and Silicosis‐MT groups exhibited a significant reduction in collagen fiber deposition and granuloma when compared to the Silicosis‐Saline group (Figures 4 and 5). Treatment with magnetized MSCs alone did not lead to this effect. No differences in collagen deposition in lung parenchyma were observed across experimental groups (Figure 4).

All groups treated with MSCs (regardless of magnetization or of MT) exhibited significantly less alveolar collapse when compared to untreated silicotic animals (Figure 4).

MT was the only treatment that significantly reduced granuloma area compared to saline control (Figures 4 and 5).

**FIGURE 6:**
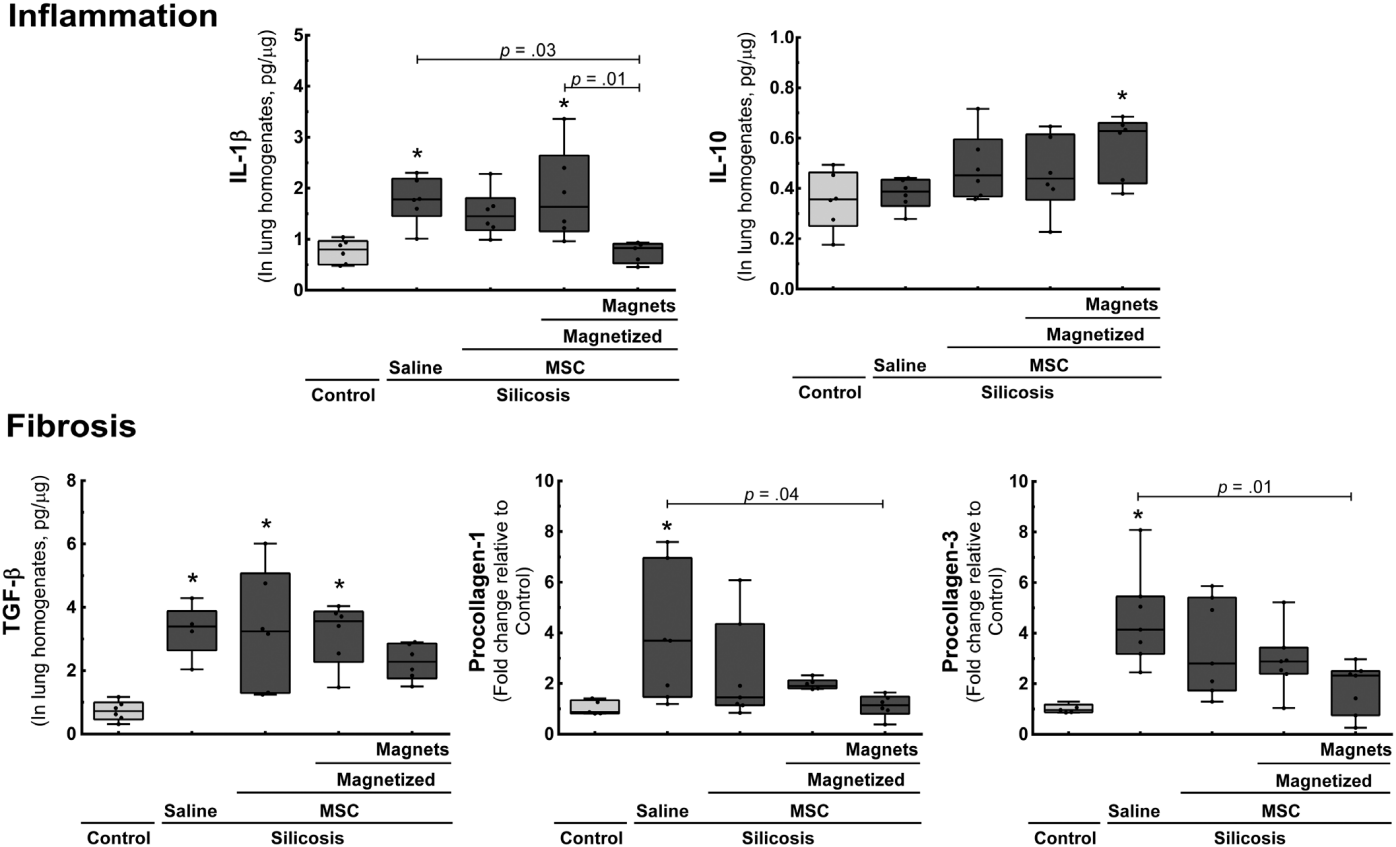
Molecular biology analysis. Protein levels of IL‐1β, IL‐10, and TGF‐β [measured by enzyme‐linked immunosorbent assay (ELISA)], as well as type I and type III procollagen mRNA expression [measured by reverse transcription followed by quantitative polymerase chain reaction (RT‐qPCR)], normalized to control (n = 6). Boxes show the interquartile range (25th‐75th percentile) while whiskers encompass the range (minimum‐maximum)

To corroborate these functional and morphological results, we measured the levels of mediators associated with inflammation and fibrosis in lung tissues (Figure 6). MT significantly reduced levels of the pro‐inflammatory cytokine IL‐1β compared to saline control, and increased levels of the anti‐inflammatory cytokine IL‐10 compared to healthy control (Figure 6). Such effects were not observed in the other experimental groups. Furthermore, levels of the fibroproliferative cytokine TGF‐β were similar in the MT and healthy control groups. Last, MT significantly reduced mRNA expression of types I and III procollagen, compared to silicotic animals treated with saline (Figure 6). The regular MSC treatment (group Silicosis‐Nonmagnetized MSC) did not exhibit this reduction.

## 5 DISCUSSION

The results obtained in this study suggest that MT of MSCs improves their therapeutic effects in experimental silicosis. After placing a pair of magnets on the chest of silicotic mice for 48 hours, there was a significant increase in the number of magnetized MSCs retained in their lungs. This higher retention was associated with more pronounced reductions in static lung elastance, resistive pressure, and granuloma.

In the context of MSC‐based therapies, MT has been used to promote more effective or focused cell delivery to areas MSCs usually do not reach after systemic administration.^23^ To date, this technique has not been well explored for the respiratory system, since most MSCs administered systemically are usually trapped in the narrow lung capillaries.^38^ In this study, however, MT was rather used to prolong MSC retention in the lungs than to improve their retention. After all, the mechanisms whereby these cells are cleared from the capillaries are still incompletely understood.^17^, ^18^ For this purpose, magnets were attached to the animals' chests for 48 hours, long enough to assess whether MT could prevent MSC removal from the lungs. To the best of our knowledge, this was the first study to use MT for this purpose.

In a previous study by our group, we observed a significant increase in iron concentration in lung homogenates from silicotic mice 48 hours after administration of magnetized MSCs and application of MT. Although this suggested higher MSC retention, its impact in therapeutic outcomes was not directly assessed.^25^ In the present study, some methodological aspects were modified in order to investigate this. First, we chose to use citrate‐functionalized maghemite (γ‐Fe_2_O_3_‐cit) nanoparticles, which can be synthesized on a large scale, are more colloidal and chemically stable, are less expensive than nanoparticles functionalized with DMSA, and are biocompatible with several cell lines, including MSCs.^29^, ^39^, ^40^ In addition, due to their negative charge, these nanoparticles do not require the use of generally toxic transfection agents for magnetizing.^23^, ^24^ Second, we used a pair of magnets, since these generate a stronger magnetic field and attract more MSCs in vitro when compared to a single magnet (data not shown). Moreover, the paired magnets were more comfortable for animals, because they are smaller in size and better fit their chest.

In the present study, magnetized MSCs co‐cultured with silica‐activated macrophages transmigrated more when exposed to the SMFs from the magnets. However, the presence of magnetic fields itself is not sufficient to explain significant transmigration (Figure 2). We then found that nanoparticle loading in combination with magnetic field exposure interferes with the expression of chemokine receptor genes, as observed in a previous report.^41^ *CCR2* and *CXCR4* are involved in MSC chemotaxis in response to MCP‐1 and SDF‐1, respectively,^42^ mRNA levels of which are increased in silicotic lungs (Figure S5). MCP‐1 is one of several chemokines secreted by silica‐activated macrophages,^43^ and is present in the plasma of silicotic patients.^44^ SDF‐1 is secreted by alveolar epithelial cells and is present in the lungs and plasma of patients with pulmonary fibrosis.^45^ The mechanisms by which magnetic fields stimulate chemokine receptor expression in magnetized MSCs remain to be elucidated. This increased expression of these chemokine receptors may contribute to the increased MSC retention observed in silicotic lungs (Figure 3), and this hypothesis should be further investigated in future studies.

Prolonged retention of MSCs in injured lungs may increase secretion of reparative factors at the site of injury. In fact, we observed increased IL‐10 levels in the lung tissue of Silicosis‐MT group animals, while there was a significant reduction in IL‐1β and TGF‐β (Figure 6). Since these mediators are associated with the progression of silica particle‐induced pulmonary fibrosis,^2^, ^46^, ^47^ this may have led to reductions in type 1 and type 3 procollagen expression (Figure 6), and to reduction in the area occupied by granulomas (Figures 4 and 5) observed in animals treated with MT.

Such attenuation of inflammation and reduction of granuloma area could be the reasons why we observed significant improvement in lung mechanics. During noninvasive analyses of pulmonary mechanics (Figure S4), Penh index increased in the Silicosis‐MT group at day 2, probably due to chest compression caused by the surgical tape jacket used to secure the paired magnets. At day 7 (ie, 5 days after magnet removal), the Penh index decreased to control‐like levels in this group.

During invasive analyses of pulmonary mechanics, only animals from the Silicosis‐MT group showed a significant reduction in static lung elastance and resistive pressure compared to silicotic animals treated with saline (Figure 4). Interestingly, the effect of treatments with nonmagnetized MSCs in this study contradicts previous findings from our group.^19^, ^22^, ^48^ This may be attributed to the type of cell used and to the timing of analysis. In a similar model of silicosis, Lopes‐Pacheco et al^48^ and de Oliveira et al^19^ reported improvement in lung mechanics after therapy with bone marrow mononuclear cells, a fraction containing not only MSCs but also other cell types. Bandeira et al also showed beneficial effects on lung function after therapy with MSCs isolated from adipose tissue,^22^ suggesting that different cell sources may lead to different therapeutic outcomes. Last, we performed invasive analysis of lung mechanics 9 days after MSC injection, while in the previous reports, this analysis was performed after 30 days.^19^, ^22^, ^48^

Nevertheless, such MT effects were not statistically different compared to those observed in Silicosis‐Nonmagnetized MSC (Figures 4 and 6). We hypothesize that the paracrine effects of MSCs would still be present at 9 days time point in these animals and that such effects tend to cease with time. Therefore, long‐term studies remain to be performed in order to assess for low long MT improves MSC retention in the lungs and whether MT could be associated with late‐phase improvement in lung function and morphometry, when compared to regular MSC treatment (eg, 15‐30 days after MSC injection).

Last, treatment with magnetized MSCs without magnet exposure did not lead to significantly reduced collagen deposition in granuloma; values in this group were different than those of animals treated with natural MSCs or with MT (Figure 4). We hypothesized that some γFe_2_O_3_ from nanoparticles could be released to the pulmonary microenvironment through EVs,^49^, ^50^ which might potentially induce inflammation and fibrosis.^51^ However, since magnetized MSC injection did not worsen collagen deposition in granuloma when compared to the silicosis‐saline group, we presume any such γFe_2_O_3_ release is not enough to be detrimental to animals, but is enough to limit MSC therapeutic efficacy. Importantly, we previously described that magnetized MSCs exposed to magnetic fields in vitro maintained associated iron in cytoplasm, unlike unexposed MSCs.^24^ This could explain why such adverse effects were not seen in the Silicosis‐MT group. In summary, potential adverse effects from γFe_2_O_3_ release must be further investigated before implementing MT in clinical practice.

## 6 CONCLUSION

In this study, we established an MSC magnetization protocol using citrate functionalized magnetic nanoparticles that kept cells viable and made them magnetically responsive. With the aid of magnets, these magnetized MSCs remained longer in the lungs, and this was associated with reduction of granuloma area and improved lung mechanics—beneficial effects for the treatment of experimentally induced silicosis in mice. Therefore, this MT technique may be a promising strategy to potentialize MSC‐based cell therapies for silicosis.

## CONFLICT OF INTEREST

D.J.W. declared research funding from United Therapeutics, Inc. The other authors declared no potential conflicts of interest.

## AUTHOR CONTRIBUTIONS

L.H.A.S.: conducted the experiments and study, contributed to data collection and analysis, interpreted the data, wrote the first draft; M.C.S., J.B.V., E.C.D.L., R.C.S.: conducted the experiments, contributed to data collection and analysis; D.J.W., M.M.M.: interpreted the data, wrote and edited the manuscript; F.F.C., P.R.M.R.: contributed to idea, conception, and design of study, interpreted the data, edited and reviewed the manuscript. All authors approved the final version of the manuscript.

7

## Supporting information


**Supplemental Figure 1** Study design and timeline. At day zero, 45 C57BL/6 mice received intratracheal saline (negative control group ‐ Ctrl) or silica (Sil). After 15 days, once changes in lung histology resembling human silicosis were established, the animals were treated with saline, non‐magnetized MSCs, or magnetized MSCs. Magnetic targeting (Sil‐MT group) was performed by holding a pair of magnets for 48 hours on the anterior chest wall of animals inoculated with magnetized MSCs. Functional evaluations were performed 2, 7, and 9 days after treatments. Lungs were harvested for further analysis at day 24. i.t., intratracheal; i.v.; intravenous.

**Supplemental Figure 2** Characterization of γ‐Fe_2_O_3_‐Citrate nanoparticles (γ‐Fe_2_O_3_‐Cit). (A) x‐ray diffraction pattern of samples. (B and C) Representative photomicrographs of transmission electron microscopy. Bars are 50 nm and 5 nm, respectively. (D) Dynamic light scattering analysis. The graph shows size distribution of γ‐Fe_2_O_3_‐Cit by intensity, when dispersed in water (red) or in cell culture medium (green), at 100 μg/mL. Three independent samples were read in triplicate (n = 9). (E) Magnetic behavior of γ‐Fe_2_O_3_‐Cit. The graph shows the magnetic moment as a function of the magnetic field, at 300 K/27°C. The saturation magnetic moment was 40.1 emu (emu)/g.

**Supplemental Figure 3** Representative photographs of MSC tracking by in vivo fluorescence imaging. Silicotic mice injected with saline solution are presented on the left of each image, while animals inoculated with magnetized MSCs (labeled with Xenolight DiR) are presented on the right. In the Sil‐MT group, a pair of magnets was secured onto the dorsal thorax of the mice immediately after intravenous injection of magnetized MSCs (0 hour time point) and kept in place for 48 hours.

**Supplemental Figure 4** Effects of magnetic targeting on lung mechanics. Non‐invasive whole‐body plethysmography. Boxes show the interquartile range, while whiskers encompass the range (minimum‐maximum) of Penh index values (n = 8). (**) Different from Control of respective time point; (†) Different from Silicosis‐Saline of respective time point; (‡) Different from Silicosis‐Non‐magnetized MSC of respective time point; (#) Different from Silicosis‐Magnetized MSC of respective time point.

**Supplemental Figure 5** Molecular biology analysis. MCP‐1 and SDF‐1 mRNA expression (measured by RT‐qPCR), normalized to control (n = 6). Boxes show the interquartile range (25th‐75th percentile), while whiskers encompass the range (minimum‐maximum).

**Supplemental table 1** Forward and reverse oligonucleotide sequences of target gene primers

**Supplemental table 2** Cell viability test by annexin/propidium iodide staining. Data refer to the mean and SD (n = 3) of the percentage of viable, early apoptotic, late apoptotic, and necrotic MSCs.

**Video S1**

## Data Availability

The data that support the findings of this study are available from the corresponding author upon reasonable request.
