# Supplementary figures and images for: Magnetic targeting increases mesenchymal stromal cell retention in lungs and enhances beneficial effects on pulmonary damage in experimental silicosis
Source: Stem Cells Transl Med. 2020 Jun 15;9(10):1244–56. doi: 10.1002/sctm.20-0004 (PMC7519769; doi:10.1002/sctm.20-0004)

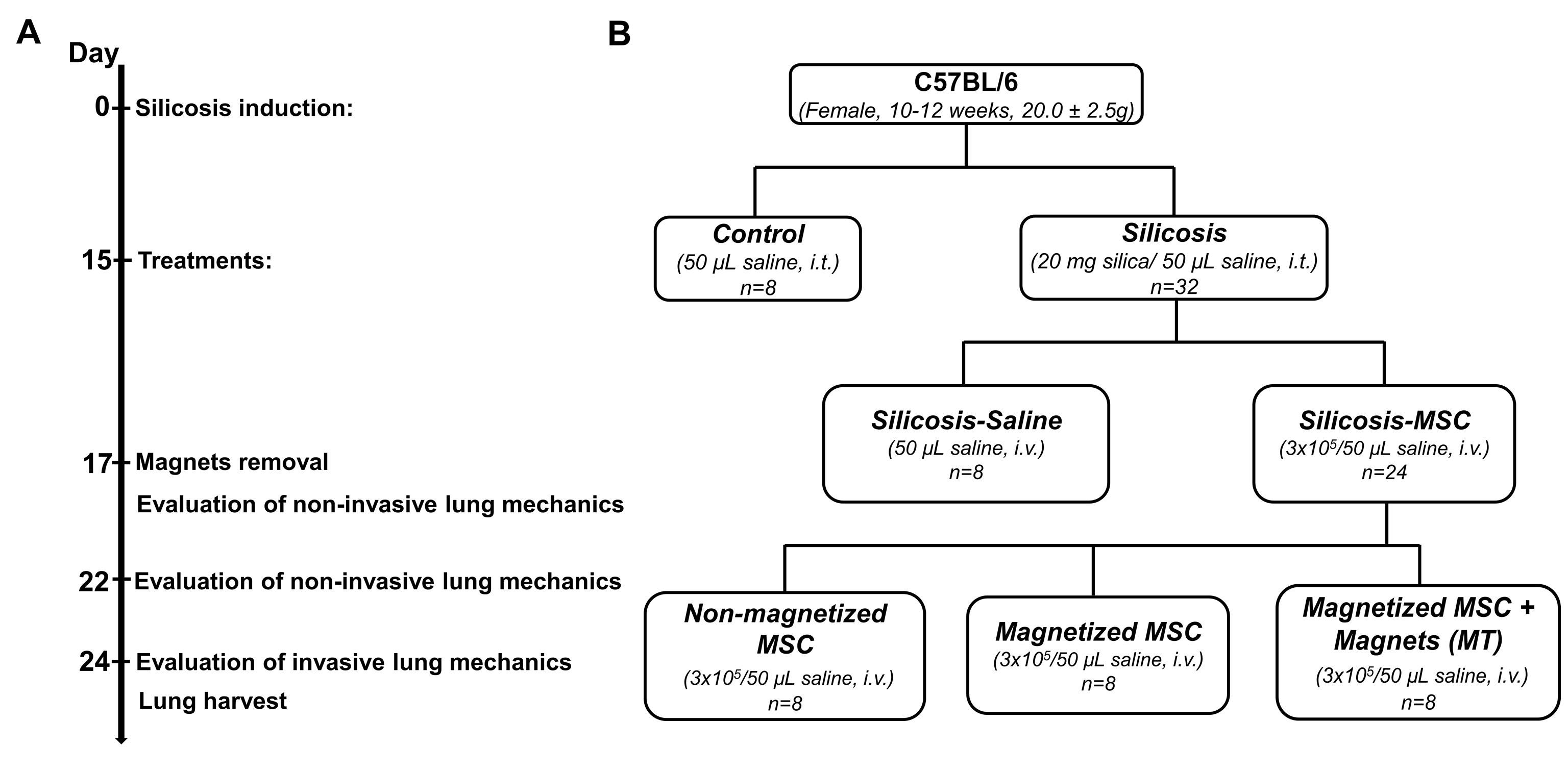

Supplement: Supplementary file 1 — Supplemental Figure 1 Study design and timeline. At day zero, 45 C57BL/6 mice received intratracheal saline (negative control group ‐ Ctrl) or silica (Sil). After 15 days, once changes in lung histology resembling human silicosis were established, the animals were treated with saline, non‐magnetized MSCs, or magnetized MSCs. Magnetic targeting (Sil‐MT group) was performed by holding a pair of magnets for 48 hours on the anterior chest wall of animals inoculated with magnetized MSCs. Functional evaluations were performed 2, 7, and 9 days after treatments. Lungs were harvested for further analysis at day 24. i.t., intratracheal; i.v.; intravenous. [file SCT3-9-1244-s001.tif]

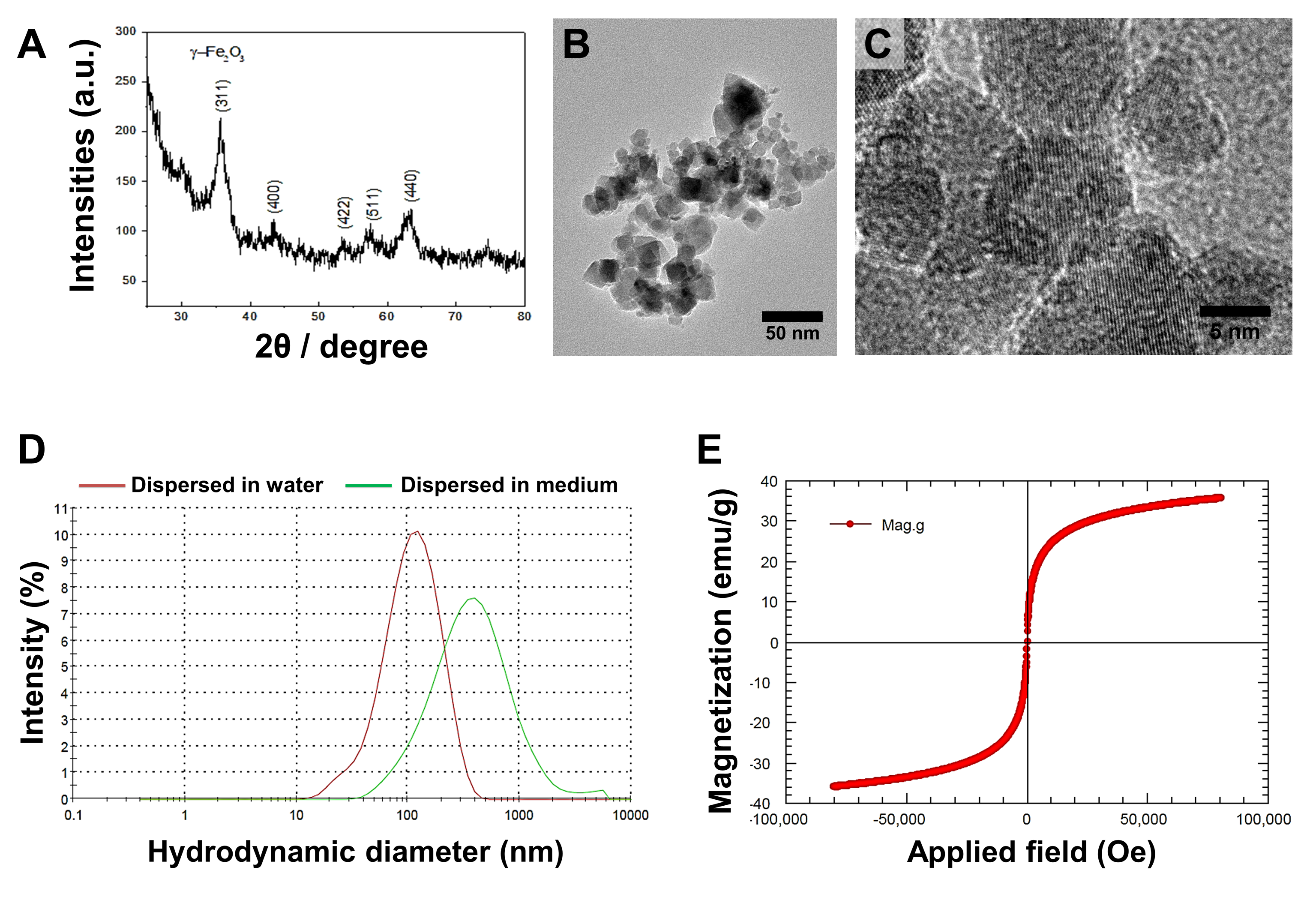

Supplement: Supplementary file 2 — Supplemental Figure 2 Characterization of γ‐Fe2O3‐Citrate nanoparticles (γ‐Fe2O3‐Cit). (A) x‐ray diffraction pattern of samples. (B and C) Representative photomicrographs of transmission electron microscopy. Bars are 50 nm and 5 nm, respectively. (D) Dynamic light scattering analysis. The graph shows size distribution of γ‐Fe2O3‐Cit by intensity, when dispersed in water (red) or in cell culture medium (green), at 100 μg/mL. Three independent samples were read in triplicate (n = 9). (E) Magnetic behavior of γ‐Fe2O3‐Cit. The graph shows the magnetic moment as a function of the magnetic field, at 300 K/27°C. The saturation magnetic moment was 40.1 emu (emu)/g. [file SCT3-9-1244-s002.tif]

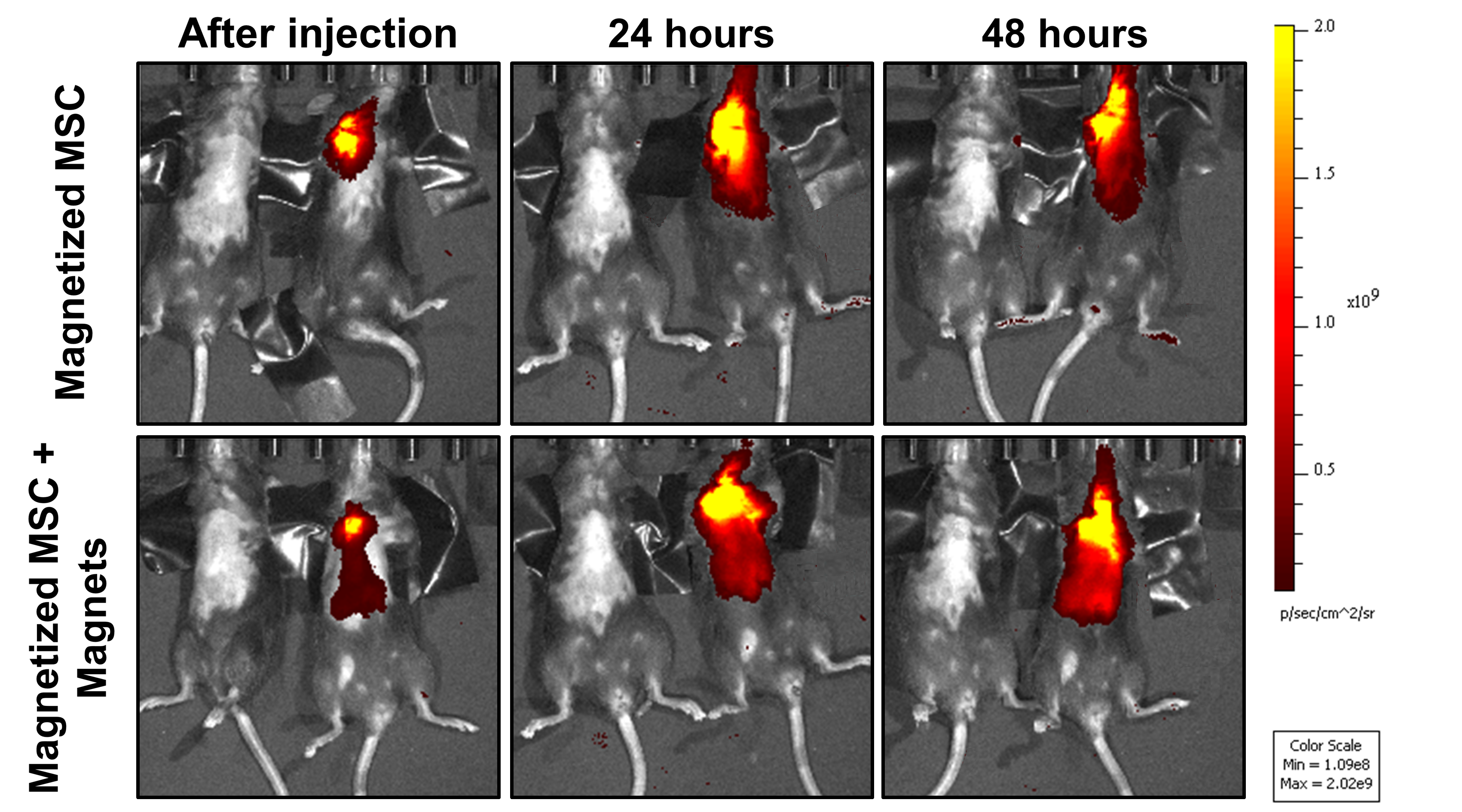

Supplement: Supplementary file 3 — Supplemental Figure 3 Representative photographs of MSC tracking by in vivo fluorescence imaging. Silicotic mice injected with saline solution are presented on the left of each image, while animals inoculated with magnetized MSCs (labeled with Xenolight DiR) are presented on the right. In the Sil‐MT group, a pair of magnets was secured onto the dorsal thorax of the mice immediately after intravenous injection of magnetized MSCs (0 hour time point) and kept in place for 48 hours. [file SCT3-9-1244-s003.tif]

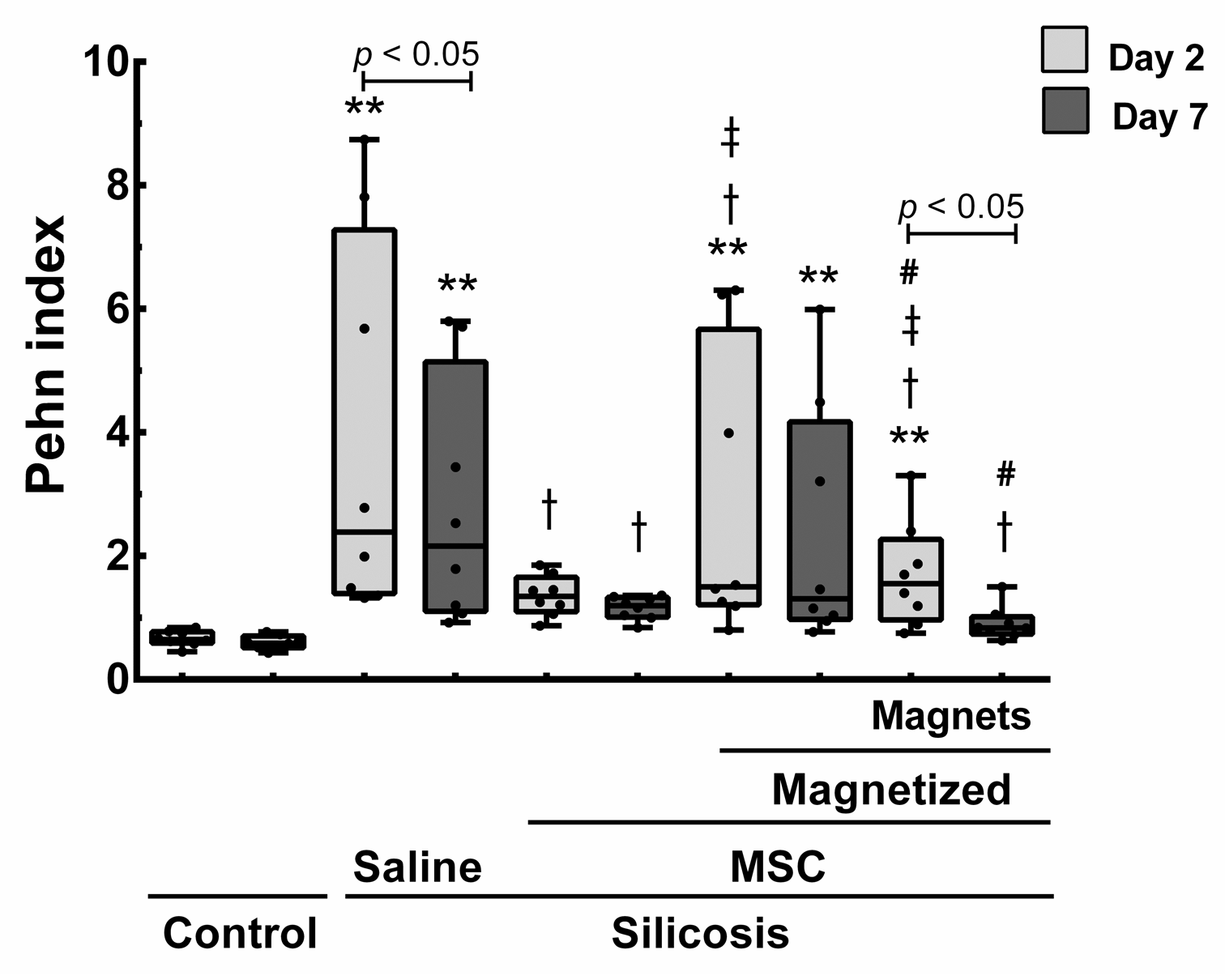

Supplement: Supplementary file 4 — Supplemental Figure 4 Effects of magnetic targeting on lung mechanics. Non‐invasive whole‐body plethysmography. Boxes show the interquartile range, while whiskers encompass the range (minimum‐maximum) of Penh index values (n = 8). (**) Different from Control of respective time point; (†) Different from Silicosis‐Saline of respective time point; (‡) Different from Silicosis‐Non‐magnetized MSC of respective time point; (#) Different from Silicosis‐Magnetized MSC of respective time point. [file SCT3-9-1244-s004.tif]

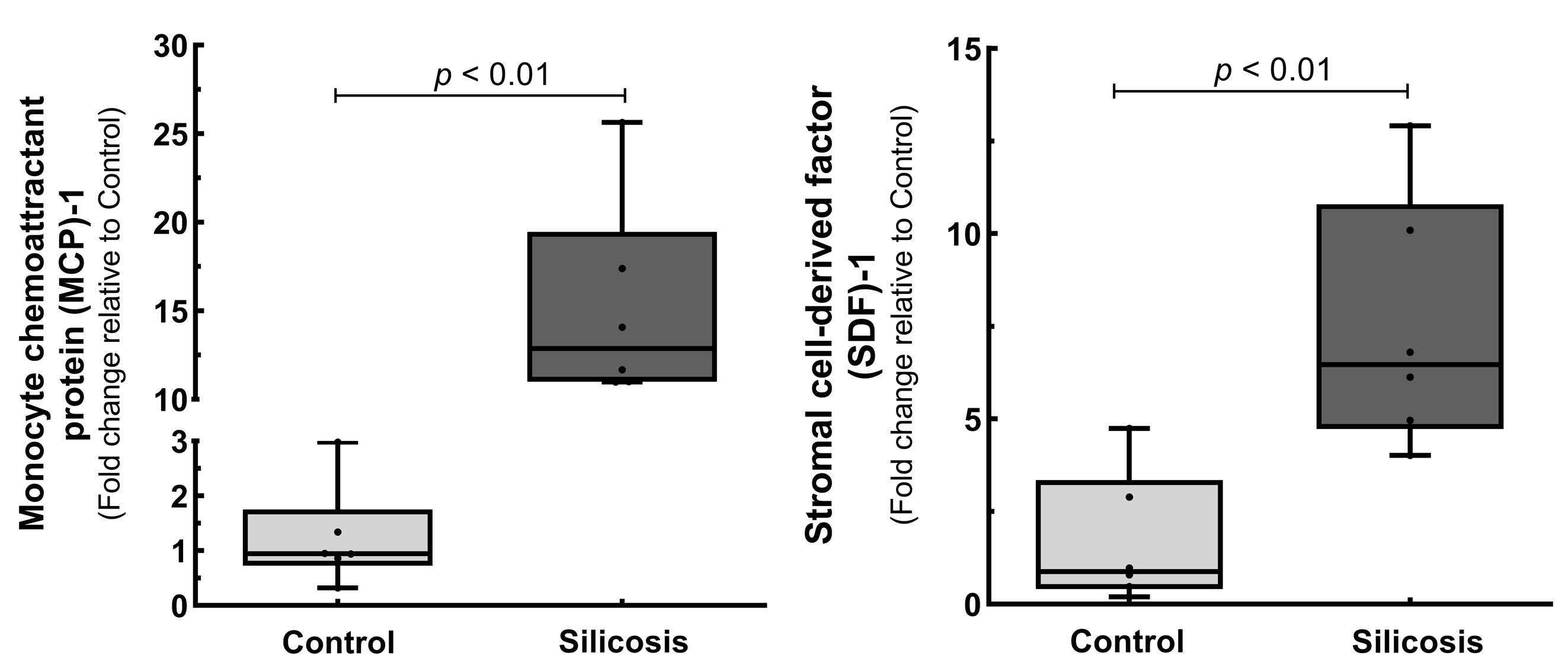

Supplement: Supplementary file 5 — Supplemental Figure 5 Molecular biology analysis. MCP‐1 and SDF‐1 mRNA expression (measured by RT‐qPCR), normalized to control (n = 6). Boxes show the interquartile range (25th‐75th percentile), while whiskers encompass the range (minimum‐maximum). [file SCT3-9-1244-s005.tif]
