# Supplementary material for: Magnetic targeting increases mesenchymal stromal cell retention in lungs and enhances beneficial effects on pulmonary damage in experimental silicosis
Source: Stem Cells Transl Med. 2020 Jun 15;9(10):1244–56. doi: 10.1002/sctm.20-0004 (PMC7519769; doi:10.1002/sctm.20-0004)
Supplement: Supplementary file 6 — Supplemental table 1 Forward and reverse oligonucleotide sequences of target gene primers [file SCT3-9-1244-s006.docx]

| **Gene** | **Forward primer** | **Reverse primer** |
| --- | --- | --- |
| *C-C chemokine receptor type 2 (CCR2)* | CCACACCCTGTTTCGCTGTA | TGGCCTGGTCTAAGTGCTTG |
| *C-X-C chemokine receptor type 4 (CXCR-4)* | CCAGCCCTCCTCCTGACTAT | CACCATCCACAGGCTATCGG |
| *Integrin α4* | CATGGGGAGAAGCTGAATGT | GGGCCTACAGAGAACAGCAG |
| *Monocyte chemoattractant protein-1 (MCP-1)* | CTTCTGGGCCTGCTGTTCA | CCAGCCTACTCATTGGGATCA |
| *Type 1 procollagen* | TGACTGGAAGAGCGGAGAGT | GTTCGGGCTGATGTACCAGT; |
| *Type III procollagen* | GTGGGACCTGGTTTCTCACCCT | GGTTGGGGCAGTCTAGTGGCTC |
| *Stromal cell-derived factor 1 (SDF-1)* | CAAGTGTGCATTGACCCGAAA | GAAGAGGGAGGAGCGAGTTAC |

**Supplemental table 1.** Forward and reverse oligonucleotide sequences of target gene primers
