# Supplementary material for: Magnetic targeting increases mesenchymal stromal cell retention in lungs and enhances beneficial effects on pulmonary damage in experimental silicosis
Source: Stem Cells Transl Med. 2020 Jun 15;9(10):1244–56. doi: 10.1002/sctm.20-0004 (PMC7519769; doi:10.1002/sctm.20-0004)
Supplement: Supplementary file 7 — Supplemental table 2 Cell viability test by annexin/propidium iodide staining. Data refer to the mean and SD (n = 3) of the percentage of viable, early apoptotic, late apoptotic, and necrotic MSCs. [file SCT3-9-1244-s007.docx]

**Supplemental table 2.** Cell viability test by annexin/propidium iodide staining. Data refer to the mean and standard deviation (*n*=3) of the percentage of viable, early apoptotic, late apoptotic, and necrotic MSCs.

|  | **Cell percentage (%)** | | | |
| --- | --- | --- | --- | --- |
| **Group** | **Viable** | **Early apoptosis** | **Late apoptosis** | **Necrosis** |
| **Non-magnetized MSCs** | 93.1 ± 0.2 | 2.0 ± 0.4 | 3.0 ± 0.1 | 1.8 ± 0.6 |
| **Magnetized MSCs** | 90.3 ± 3.4 | 4.6 ± 0.9 | 2.4 ± 1.9 | 2.7 ± 0.8 |
